# The relationship between skeletal muscle mass and cognitive impairment in older adults: a longitudinal study based on CLHLS

**DOI:** 10.3389/fpubh.2025.1571510

**Published:** 2025-05-26

**Authors:** Shani Feng, Kaixuan Xie, Yu Zhang, Xinyu Yang, Youpeng Guo, Dandan Xu

**Affiliations:** ^1^The Second People's Hospital of Yuhuan, Yuhuan, China; ^2^Department of Nursing, College of Medical Science, Huzhou University, Huzhou, China

**Keywords:** skeletal muscle mass, older adult, cognitive impairment, restricted cubic spline, longitudinal study

## Abstract

**Objective:**

To explore the relationship between skeletal muscle mass and cognitive impairment in older adults.

**Methods:**

Based on the longitudinal survey data of the four phases of the China Older Adults Health Influencing Factors Longitudinal Survey Project from 2008 to 2018, this study used the Cox regression analysis method and the R4.3.3 software to construct a limiting cubic spline model to deeply explore the dose–response relationship between skeletal muscle mass and cognitive impairment in the older adults.

**Results:**

In this study, 2,199 older adults (with ages ranging from 61 to 108 years, 51.7% of whom are female and 48.2% are male) were included and followed up for a decade, during which four rounds of data collection were carried out. The results showed that the proportion of new-onset cognitive impairment in men was 14.61%, while the proportion in women was 33.48%. After adjusting for confounding factors, Cox regression results showed that the larger the muscle mass group, the greater the protective effect on cognitive impairment. The results of RCS showed that the association between skeletal muscle mass and cognitive impairment in the older adults group (*P*_overall trend_ <0.05, *P*_nonlinear_ <0.05) showed a nonlinear increasing trend, and there were gender differences. ASMI < 7.2 kg/m^2^ in older men was a risk factor for cognitive impairment (*HR*>1); ASMI < 5.4 kg/m^2^ in older women was a risk factor for cognitive impairment (*HR*>1). This suggests that ASMI should be maintained at a high level in older men and women, respectively.

**Conclusion:**

There is a link between skeletal muscle mass and cognitive impairment in older adults, and cognitive function can be improved through early intervention or by improving the level of skeletal muscle mass in older adults.

## Introduction

With the deepening of the aging society, the spread of chronic diseases not only exacerbates the burden on the medical system but also poses a severe challenge to the overall health security system of society. Among them, the cognitive function problems the older adults face are becoming increasingly prominent, which has become a public health challenge that cannot be ignored (1, 2). Cognitive impairment is a precursor to the development of dementia, which is mainly manifested in the loss of memory, behavior, and daily living behaviors. More than 35 million people worldwide are already living with dementia, and the number of people living with dementia is predicted to reach 131.5 million by 2050 (3).

According to the latest statistics, the prevalence of cognitive impairment among the older adults in China is as high as 15.5% and more than 50% of these patients will develop irreversible Alzheimer’s disease or other forms of dementia within the next 4 to 6 years, which will put great pressure on the public health system (4, 5). Therefore, early research and intervention on cognitive impairment is crucial.

Cognitive dysfunction is primarily manifested in the accumulation of substantial amyloid-*β* protein in the brains of older adult individuals, accompanied by the loss of a large number of neurons, leading to a neurodegenerative disease (6). However, cognitive function is related to numerous factors, including exercise (7), physical frailty (8), and muscle mass (9, 10). Among them, the decline in muscle mass can reduce the levels of trophic factors in the brain, upregulate the ubiquitin-dependent proteolytic system, result in amyloidosis, and ultimately progress to dementia (11). However, Gabor (12) argues that cognitive function is not associated with surgical sarcopenia, regardless of how it is defined, regardless of the way it is defined. Levine (13) reported that sarcopenia in older adults aged 60–69 years was not associated with cognitive function. Some studies confirm that confirm that muscle mass levels are associated with cognitive function between different sexes (12, 14). However, Auyeung (15) reported no difference in a four-year follow-up between men and women over 65 years of age with normal baseline cognition. These differences may be due to bias in the lack of randomization of samples, inconsistency in the definition of sarcopenia, and limitations in cross-sectional studies (12, 13, 15).

In addition, studies have shown that cognitive function varies over time (16, 17), and the strength of the association between skeletal muscle mass and cognitive function changes over time. China has the largest aging population, and the temporal association between skeletal muscle mass and cognitive function has not been studied in national cohort studies.

In this study, muscle mass was assessed using the appendicular skeletal muscle mass index (ASMI). Compared to traditional BMI, ASMI demonstrates superior advantages as it directly quantifies muscle mass and serves as the core diagnostic criterion for sarcopenia. In contrast, BMI merely reflects the weight-to-height ratio without distinguishing between fat and muscle mass. Notably, elevated BMI may mask low muscle quality, which is associated with significantly elevated cognitive risks in such populations. ASMI provides a more precise characterization of the muscle-cognition relationship. Evidence from the Health and Retirement Study (HRS) indicates that baseline low ASMI predicts cognitive decline over 5 years (HR = 1.32), whereas BMI shows no predictive value. Therefore, this study employs ASMI as the standard metric for evaluating skeletal muscle quality.

Therefore, this study used the Chinese Longitudinal Health and Longevity Survey (CLHLS) database to explore the dose–response relationship between related factors and cognitive impairment by using the Restricted cubic spline (RCS) method, aiming to provide a novel measurement method to evaluate skeletal muscle mass. To explore the strength of its temporal association and to provide a scientific basis for the prevention and health intervention strategies of cognitive impairment.

## Methods

### Data sources and study population

The CLHLS database was established in 1998 by the Center for Healthy Aging and Development of Peking University and the National Academy of Development and is currently the largest cohort study for the older adults in China (18). The aim is to provide a representative survey of the older adults over 65 years of age, covering 23 provinces, municipalities, and autonomous regions across the country. CLHLS received ethical approval from the Biomedical Ethics Committee of Peking University in China (IRB00001052-13074).

**Figure 1:**
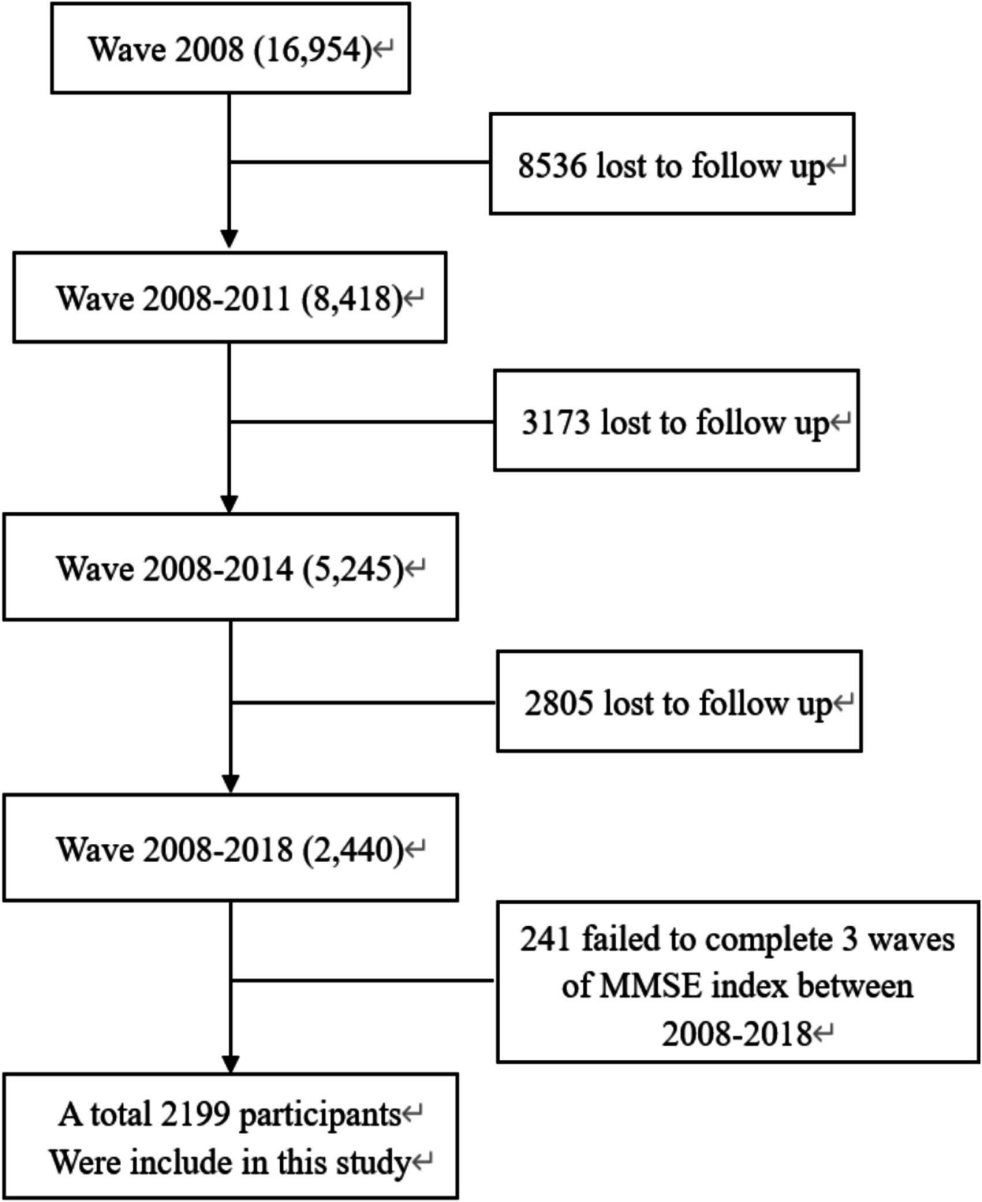
Flowchart of the study population.

In this study, 2,199 older adult individuals aged 61–108 years participated in four surveys conducted in 2008, 2011, 2014, and 2018. The participants included 48.2% males and 51.7% females. All data were sourced from the CLHLS database, excluding samples with cognitive impairment at baseline and those who were unable to answer the scale questions or had missing key variables (Figure 1).

### Research methods

#### Measurement of cognitive function

The database evaluates the cognitive function of the older adults through the Chinese version of the Mini-Mental State Examination (MMSE), which consists of 4 dimensions and a total of 24 items, specifically covering general ability, reaction ability, attention and calculation ability, recall ability, and language comprehension. 1 point for correct answers, 0 points for incorrect answers, the total score range is 0 ~ 30 points, the higher the score, the better the cognitive function of the older adults. Based on the educational level of the subjects, those with a score of ≤ 17 for illiterates, ≤20 for those with a primary school education, and ≤24 for those with a junior high school education are defined as having cognitive impairment (19).

#### Muscle mass assessment

In this study, the ASMI, adjusted for height, was used to assess skeletal muscle mass. The formula for calculating ASMI is ASMI = ASM/height^2^ (where height is in meters). Among them, Appendicular Skeletal Muscle Mass (ASM) is a key indicator for measuring skeletal muscle mass levels in the human body and is often used in the definition of sarcopenia (20). The formula for calculating ASM is ASM = 0.193 * body weight (Kg) + 0.107 * height (cm)—4.157 * gender (male = 1, female = 2)—0.037 * age (years)—2.631. This indicator is highly consistent with the measurements obtained by Dual-energy X-ray Absorptiometry (DXA). However, DXA is costly and involves relatively longer radiation exposure (21). Therefore, ASMI has been adopted as a more economical and convenient indicator for research purposes.

#### Covariates

Taking into account the influence of confounding factors, the covariates include age (years), type of residence (urban or rural), living status (living alone or not), marital status (married, divorced, widowed, etc.), educational attainment (illiteracy, primary school, junior high school, high school, etc.), smoking status (yes or no), alcohol consumption status (yes or no), exercise (yes or no), and BMI (underweight, normal, and obese). BMI is calculated based on the subject’s height and weight, using the formula BMI = weight (kg) / height^2^ (m^2^).

### Statistical methods

The mean ± standard deviation ( $\bar{x}$ ± s) was used for the measurement data. The *t*-test was used for differences between groups. The frequency and percentage of the counting data were described, and the *x^2^* analysis was used to analyze the differences. Among them, ASMI was divided into quartile groups, and the Cox proportional-hazards regression model was used to compare the temporal effects of each variable on cognitive impairment, and the dose–response relationship between ASMI and cognitive impairment was analyzed by RCS, and the Akaike Information Criterion (AIC) was employed to evaluate the goodness-of-fit and complexity of the restricted cubic spline (RCS) models. A lower AIC value indicates a better balance between model simplicity and fitting capability. In RCS models, the AIC can be used to select the optimal number or placement of knots, thereby avoiding overfitting of nonlinear relationships. All statistical analyses were conducted using SPSS 27.0 and R4.4.3 software, and the significance level of statistical tests was 0.05.

## Results

### Sample descriptive analysis

Among the 2,199 study subjects, 1,061 (48.25%) were males and 1,138 (51.75%) were females, and after 10 years of follow-up, 155 (14.61%) were older adult men with new cognitive impairment and 381 (33.48%) were females with new cognitive impairment. Among them, the proportion of cognitive impairment in the low ASMI group was the highest (44.03%). There were statistically significant differences in age, residence, pension, degree, marriage, smoking, alcohol consumption, exercise, BMI, and ASMI between the two groups (*p* < 0.05), and the specific data are detailed in Table 1.

**Table 1:** Baseline characteristics of the study population (*n* = 2,199).

Characteristics		Cognitive impairment ( $\bar{x}$ ± s / %)		*p*
		No	Yes	
Age (year)		72.98 ± 7.17	78.88 ± 8.79	<0.001
Gender	Male	906(54.48)	155(28.92)	<0.001
	Female	757(45.52)	381(71.08)	
Residence	City	235(14.13)	54(10.07)	<0.001
	Towns	337(20.26)	91(16.98)	
	Countryside	1,091(65.6)	391(72.95)	
Pension	With family	1,425(85.69)	432(80.6)	<0.001
	Alone	227(13.65)	100(18.66)	
	Older adult care institutions	11(0.66)	4(0.75)	
Degree	Illiterate	621(37.34)	378(70.52)	<0.001
	Primary school education	731(43.96)	133(24.81)	
	Junior high school education	275(16.54)	24(4.48)	
	High school education and above	36(2.16)	1(0.19)	
Marriage	Married	1,128(67.83)	261(48.69)	<0.001
	Divorce	4(0.24)	1(0.19)	
	Widowed	514(30.91)	270(50.37)	
	Unmarried	17(1.02)	4(0.75)	
Smoke	No	1,227(73.78)	458(85.45)	<0.001
	Yes	436(26.22)	78(14.55)	
Drink	No	1,253(75.35)	448(83.58)	<0.001
	Yes	410(24.65)	88(16.42)	
Exercise	No	1,038(62.42)	377(70.34)	<0.001
	Yes	625(37.58)	159(29.66)	
ASMI (kg/m^2^)	<5.26	317(19.06)	236(44.03)	<0.001
	5.26–6.40	408(24.53)	140(26.12)	
	6.40–7.20	450(27.06)	101(18.84)	
	>7.20	488(29.34)	59(11.01)	
BMI (kg/m^2^)	<18.5	299(17.98)	161(30.04)	<0.001
	18.5–24	975(58.63)	281(52.43)	
	>24	389(23.39)	94(17.54)	

### Cox regression analysis between ASMI and cognitive impairment

The Cox proportional hazards regression model was performed with cognitive impairment (no = 0, yes = 1) as the outcome variable, ASMI as the independent variable, and place of residence, age, living environment, smoking, drinking, exercise, education, marital status, BMI, etc. as covariates. After adjusting for covariates, age, gender, education, and ASMI were the factors influencing cognitive impairment. Increasing age was a risk factor for cognitive function (*HR*>1, *p* < 0.05), female was a risk factor for cognitive function (*HR*>1, *p* < 0.05), and high education was a protective factor for cognitive function (*HR*<1, *p* < 0.05). Higher levels of ASMI were protective of cognitive function (*HR*<1, *p* < 0.05). They were shown in Table 2.

**Table 2:** Multivariable cox proportional hazards regression analysis of cognitive impairment.

	*β*	*SE*	*p*	*HR* (95%*CI*)
Age (year)	0.048	0.005	<0.01	1.049(1.038–1.06)
Gender (Male)	0.23	0.188	<0.01	1.258(1.014–1.502)
Residence (City)				1
Towns	−0.039	0.178	0.824	0.961(0.678–1.362)
Countryside	0.094	0.157	0.551	1.098(0.807–1.494)
Pension (With Family)				1
Alone	−0.094	0.123	0.448	0.911(0.715–1.16)
Older adult care institutions	−0.187	0.517	0.717	0.829(0.301–2.286)
Degree (Illiterate)				1
Primary school education	−0.583	0.112	<0.01	0.558(0.448–0.696)
Junior high school education	−0.997	0.225	<0.01	0.369(0.237–0.573)
High school education and above	−2.283	1.01	0.024	0.102(0.014–0.739)
Marriage (Married)				1
Divorce	0.675	1.008	0.503	1.963(0.272–14.158)
Widowed	−0.041	0.109	0.708	0.96(0.775–1.189)
Unmarried	0.155	0.515	0.763	1.168(0.426–3.206)
Smoke (No)	−0.119	0.139	0.39	0.887(0.676–1.165)
Drink (No)	0.043	0.128	0.736	1.044(0.813–1.341)
Exercise (No)	−0.074	0.102	0.464	0.928(0.761–1.133)
BMI (<18.5; kg/m^2^)				1
18.5–24	−0.107	0.104	0.303	0.899(0.734–1.101)
>24	−0.091	0.146	0.533	0.913(0.685–1.216)
ASMI (<5.26; kg/m^2^)				1
5.26–6.40	−0.139	0.12	0.245	0.87(0.688–1.1)
6.40–7.20	−0.199	0.192	0.299	0.819(0.563–1.193)
>7.20	−0.5	0.238	0.036	0.606(0.38–0.967)

References are in parentheses.

### Dose–response relationship

**Figure 2:**
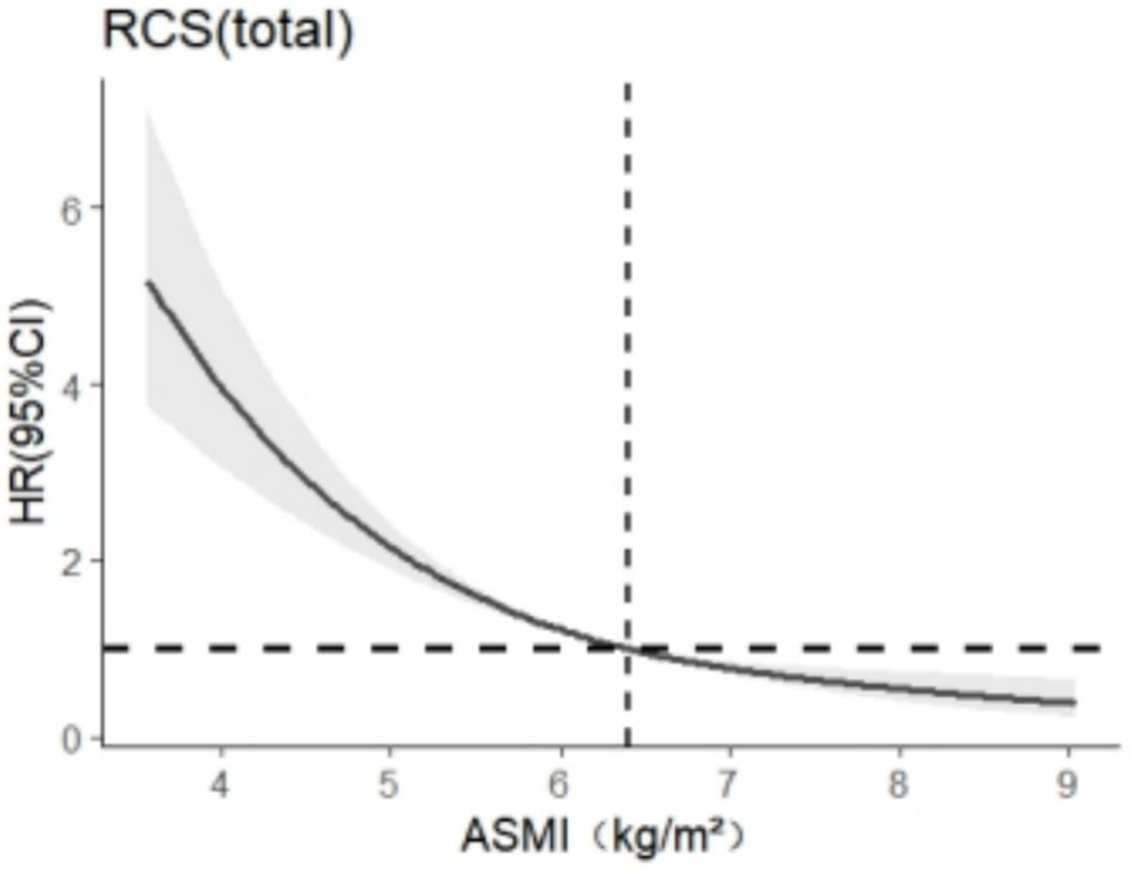
Dose–response relationship between skeletal muscle mass and cognitive impairment in the older adult population.

The RCS model was used to analyze the relationship between skeletal muscle mass and cognitive impairment in the older adults group, and 3 ~ 7 models were selected as appropriate, the abscissa was the continuous change of skeletal muscle mass, and the ordinate was the corresponding predicted value (*HR*), in which the shaded part represented 95%*CI*. According to the *AIC* criterion and the spline regression coefficient, it was found that the total *AIC*_population_ = 7913.301 reached the minimum in the model, and the number of nodes was 5, as shown in Figure 2. There was a nonlinear dose–response relationship between skeletal muscle mass and cognitive impairment in the total older adult population (*P*_total trend_ <0.05, *P*_nonlinearity_ < 0.05), and when skeletal muscle mass <6.4 kg/m^2^, *HR* >1, suggests that skeletal muscle mass in this range increases the risk of cognitive impairment in the older adults.

**Figure 3:**
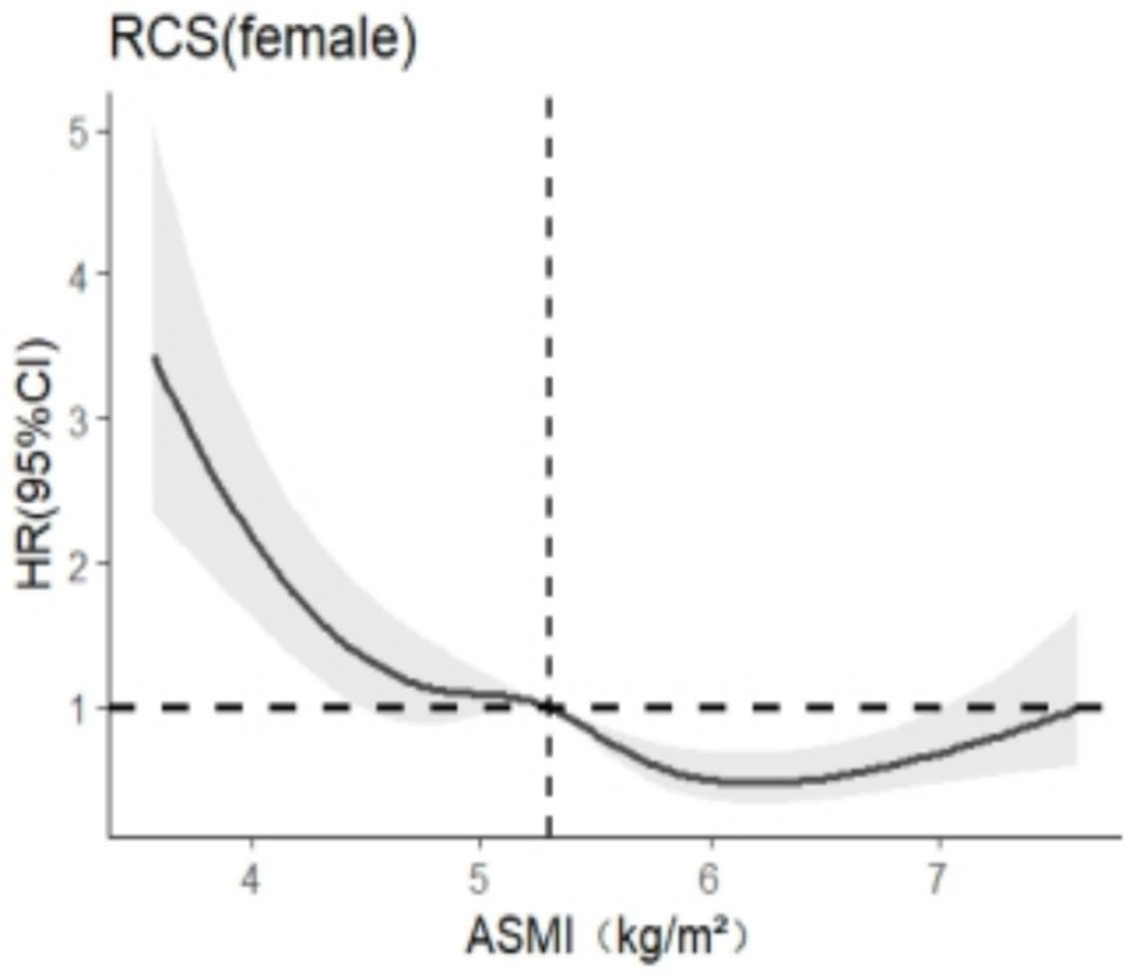
Dose–response relationship between skeletal muscle mass and cognitive impairment in females.

**Figure 4:**
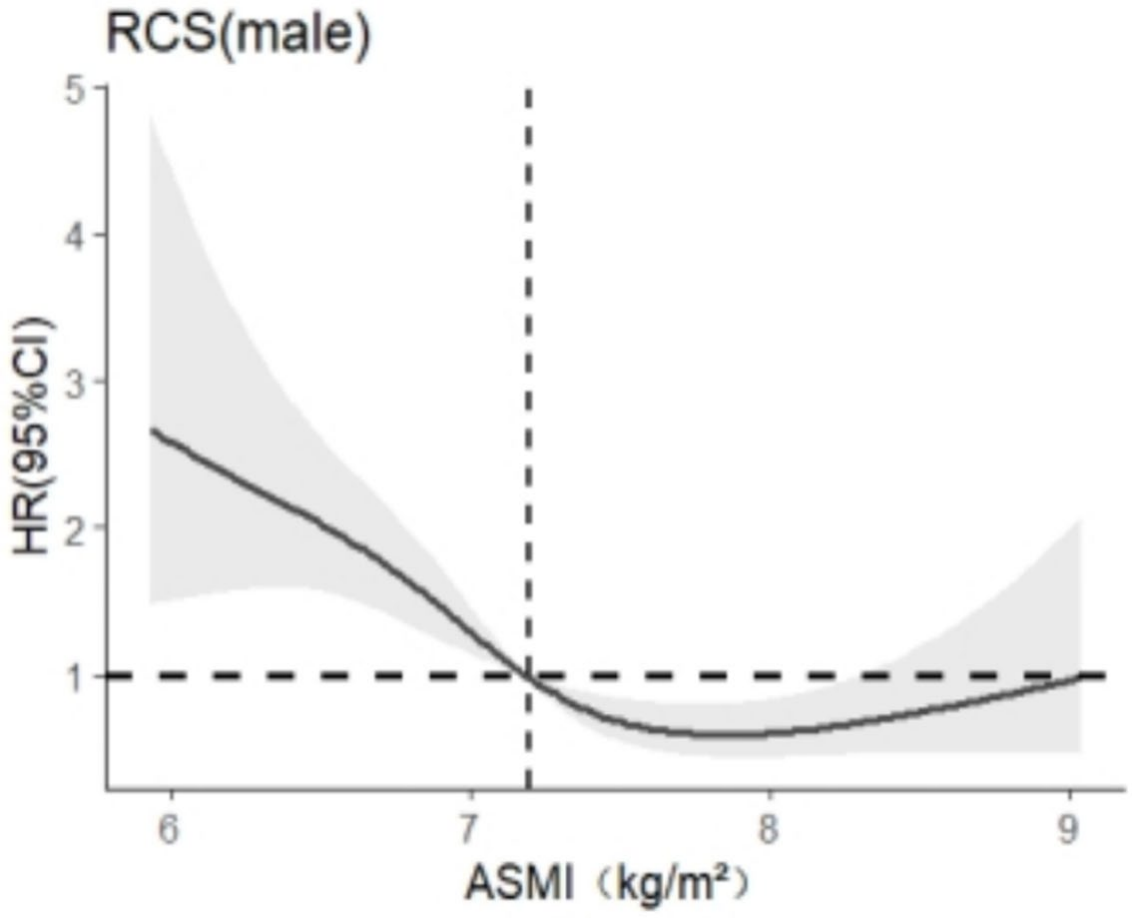
Dose–response relationship between skeletal muscle mass and cognitive impairment in males.

After stratification by sex, RCS was used to analyze the relationship between skeletal muscle mass and cognitive impairment. The results are shown in Figures 3, 4. In this study, we found that among the models related to cognitive impairment, *AIC*_elderly male_ = 2106.909 and *AIC*_aged female_ = 5140.588 reached the smallest, with 5 nodes and 5 nodes, respectively. After adjusting for the related confounding factors, it was found that there was a nonlinear dose–response relationship between skeletal muscle mass and cognitive impairment in older adult men (*P*_total trend_ <0.05, *P*_nonlinear_ <0.05), and when skeletal muscle mass <7.2 kg/m^2^, *HR* >1 suggests that skeletal muscle mass is a risk factor for cognitive impairment in older adult men, There was a nonlinear dose–response relationship between skeletal muscle mass and cognitive impairment in older adult women (*P*_total trend_ <0.05, *P*_nonlinearity_ < 0.05), and when skeletal muscle mass <5.4 kg/m^2^, *HR* >1, indicating that skeletal muscle mass was a risk factor for cognitive impairment in older adult women at this time.

### Subgroup analyses

**Figure 5:**
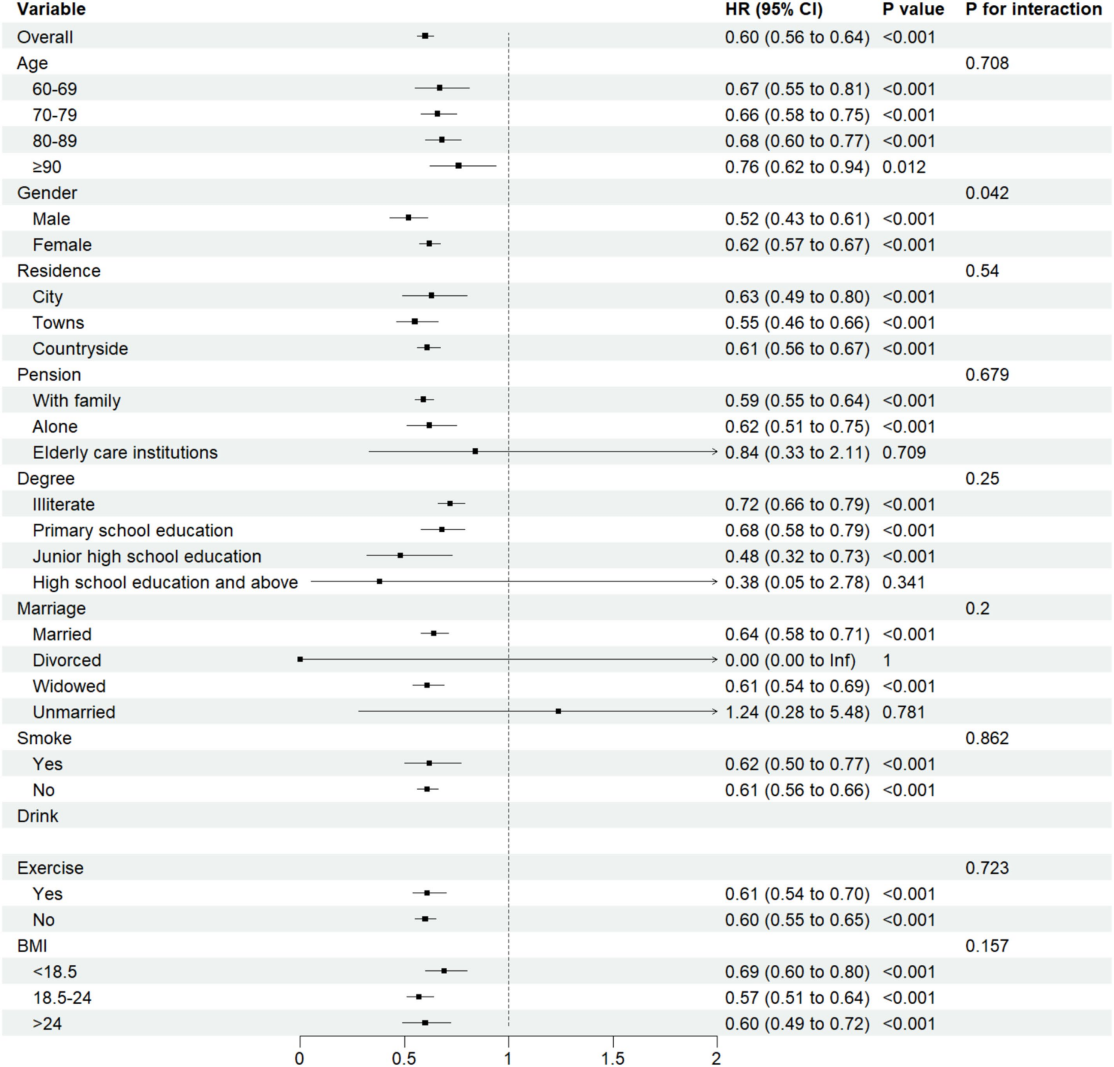
Subgroup analyses for ASMI and cognitive impairment interaction.

We conducted subgroup analyses based on gender, age, residence, pension, degree, marriage, smoke, drink, exercise, and BMI. Among these analyses, a significant interaction was observed between ASMI and cognitive impairment in the gender subgroup (*P*_for interaction_ = 0.042). No significant interactions (*p* > 0.05) were detected in other subgroups. Detailed results are presented in Figure 5.

## Discussion

With age, the risk of developing dementia shows a gradual upward trend, and its early signs often manifest as a decline in cognitive function. In this study, a total of 536 cases of newly emerged cognitive impairment were identified among the older adults, with an incidence density of 2.43/100 person-years, which is generally consistent with the incidence density of 2.67/100 person-years reported by Yifu Liang in a 5-year follow-up of the CLHLS (22). Furthermore, Cox regression analysis revealed that women are more susceptible to cognitive impairment than men, with a risk that is 1.258 times higher than that of men. This finding is consistent with the conclusion drawn by Nebel (23). The underlying reason may be that women are more prone to exposure to risk factors for cognitive impairment compared to men, such as depression and memory decline. After women enter menopause, their estrogen levels drop sharply, making them more susceptible to depression (24, 25), which in turn increases their risk of developing cognitive impairment. Moreover, estradiol-related hippocampal impairment leads to a decline in women’s memory (26), which also increases their risk of developing the disease. Therefore, exploring the relationship between cognitive function and muscle mass separately by gender is of great significance for early intervention in cognitive impairment.

RCS indicates that as ASMI decreases, the strength of its association with the risk of cognitive impairment increases nonlinearly. This phenomenon may be attributed to the reduction in physical activity and the dysregulation of myokine secretion in older adult individuals as they age, leading to a decline in skeletal muscle mass (27, 28). This decline further affects the local levels of trophic factors in the brain and may trigger mitochondrial oxidative stress, systemic inflammation, as well as upregulation or impairment of the ubiquitin-dependent proteolytic system (UPS) (29). As the primary mechanism for clearing short-lived, damaged, and misfolded nuclear and cytosolic proteins, upregulation or impairment of the UPS can lead to abnormal cellular metabolism and the accumulation of toxic substances in the brain, thereby triggering amyloidogenesis and ultimately adversely affecting cognitive function (30).

The subgroup analysis revealed significant gender-based differences in the association strength between ASMI and cognitive impairment (*P*_for interaction_ = 0.042).Compared to males, females derived weaker cognitive protective effects from increased muscle mass, suggesting that women should maintain higher levels of muscle mass to preserve favorable cognitive function. And RCS found that ASMI should be maintained at 7.2 kg/m^2^ or above for older adult men and 5.4 kg/m^2^ or above for older adult women. This finding is consistent with the results of Taichi (31) and Yasuyuki (32). This may be because muscle loss predisposes to a decrease in hepatic insulin-like growth factor-1 (IGF-1) levels (33), which adversely affects neuronal signaling, nerve nutrient supply, and neuroprotection. Therefore, the decline of IGF-1 can affect the activity of neurons, leading to amyloidosis, cognitive deficits, loss of synaptic vesicle protein, and ultimately the development of dementia (34). The primary reason for the gender difference lies in the fact that changes in IGF-1 levels have a more significant impact on women compared to men (35). This phenomenon may arise because IGF-1 and estrogen have a synergistic effect, aiding women in maintaining skeletal muscle mass (36, 37). However, after menopause, women experience a decline in circulating growth hormone and IGF-1 levels, which fails to sustain muscle mass levels, accelerating muscle loss. In contrast, this phenomenon is not as pronounced in men. The muscle mass of men is primarily maintained through the regulation of somatostatin, whereas the decline in IGF-1 levels has a relatively limited impact on men’s muscle mass compared to women (38). Therefore, it is recommended that older adult women should pay more attention to their diet and physical exercise to improve muscle mass and prevent the occurrence of dementia.

In the realm of gender-specific thresholds, males with a higher baseline muscle mass exhibit a greater reliance on the absolute quantity of muscle for actin secretion, with a critical threshold of 7.2 kg/m^2^. Below this threshold, there is a significant decrease in IGF-1 levels, which adversely affects neuroprotective functions (35). In contrast, females, despite generally having lower muscle mass, benefit from the synergistic enhancement of IGF-1 efficacy by estrogen (37). However, postmenopausal estrogen depletion diminishes IGF-1’s regulatory capacity in both muscular and neural systems, suggesting that even ASMI levels above the male-specific threshold may be inadequate for maintaining cognitive function (36). This study, utilizing ASMI formulas derived from the CLHLS, reveals lower thresholds in Chinese populations compared to Western data, potentially due to lifestyle disparities, genetic predispositions, and a higher prevalence of chronic diseases, particularly in rural regions. These thresholds closely mirror the Asian sarcopenia consensus values determined by machine-measured ASMI (21), yet they are still below the Western benchmarks (39). Cross-cultural validation is supported by Japanese studies that identify elevated cognitive impairment risks at female ASMI levels below 5.5 kg/m^2^, aligning with Chinese thresholds (31). The Framingham cohort also associates ASMI levels below 7.0 kg/m^2^ in males and 5.7 kg/m^2^ in females with cognitive decline, indicating population-dependent variations in threshold specificity (40).

However, Jiang Dian (41) found no dose–response relationship between ASMI and cognitive decline in a cross-sectional study of 4,184 older adult individuals, which is inconsistent with the findings of this study. The reasons for this discrepancy may lie in the fact that Jiang Dian’s study employed a cross-sectional design, examining only the population at a single point in time in 2011, thus yielding weaker causal inferences. Additionally, the definition of cognitive decline in older adult individuals in that study relied solely on self-reports, which may introduce bias and affect the accuracy of the results. Furthermore, Zhang Jiajia (42) suggests a bidirectional association between sarcopenia and cognitive function. This study complements and refines that understanding, thus possessing great value for public health guidelines.

This study has several advantages. First, it utilizes the RCS model to explore the relationship between skeletal muscle mass and cognitive function in greater detail, providing more valuable insights. Second, this study adopts a new method for measuring skeletal muscle mass that is simpler and more cost-effective compared to the DXA method commonly used in most studies. Third, this study employs a representative database of older adult Chinese individuals and has a longer follow-up period. However, it also has corresponding limitations. Although this study is a longitudinal study based on big data, it still lacks direct biological indicators related to muscle mass, a limitation that needs to be addressed in future research. Therefore, further experimental studies are necessary to validate and deepen this perspective.

## Limitations and prospects

This study is limited by the inherent design of the CLHLS database, with notable shortcomings. Firstly, the research population consists exclusively of Chinese older adults, which compromises the universality of the thresholds. Secondly, the absence of relevant biological information and imaging data in the database may introduce diagnostic bias, as the findings lack the precision of clinical diagnoses. Thirdly, the habitual physical activity of participants, a significant specific lifestyle factor influencing both sarcopenia and cognitive function, was not incorporated into the analysis. Future studies should employ more rigorous methodologies to improve scientific rigor. Incorporating habitual physical activity as a factor would strengthen the paper’s arguments and offer valuable insights for researchers in this field.

## Conclusion

This study found that in the general older adult population when ASMI falls below 6.4 kg/m^2^, specifically in older adult men with ASMI below 7.2 kg/m^2^ and older adult women with ASMI below 5.4 kg/m^2^, skeletal muscle mass becomes a risk factor for cognitive impairment. Therefore, in the context of China’s rapidly aging population, it is particularly crucial and forward-thinking to prioritize and closely monitor skeletal muscle mass in the older adults to prevent cognitive impairment.

## Data Availability

Publicly available datasets were analyzed in this study. This data can be found at: The data that support the findings of this study are openly available at: https://opendata.pku.edu.cn/dataverse/CHADS.
